# Insulin-like growth factors 1 and 2 regulate gene expression and enzymatic activity of *cyp17a1* in ovarian follicles of the yellowtail, *Seriola quinqueradiata*

**DOI:** 10.1016/j.heliyon.2020.e04181

**Published:** 2020-06-10

**Authors:** Kentaro Higuchi, Yukinori Kazeto, Yuichi Ozaki, Daisuke Izumida, Takuro Hotta, Kiyoshi Soyano, Koichiro Gen

**Affiliations:** a Seikai National Fisheries Research Institute, Japan Fisheries Research and Education Agency, Taira-machi, Nagasaki 851-2213, Japan; b Kamiura Station, National Research Institute of Aquaculture, Japan Fisheries Research and Education Agency, Kamiura, Saiki, Oita 879-2602, Japan; c National Research Institute of Aquaculture, Japan Fisheries Research and Education Agency, Tamaki, Mie 519-0423, Japan; d Institute for East China Sea Research, Nagasaki University, Taira-machi, Nagasaki 851-2213, Japan; e Goto Station, Seikai National Fisheries Research Institute, Japan Fisheries Research and Education Agency, Tamanoura, Goto, Nagasaki 853-0508, Japan

**Keywords:** Cell biology, Molecular biology, Physiology, Aquaculture, Cell culture, Reproductive hormone, Steroid hormones, Animal physiology, Insulin-like growth factor, Ovary, Steroidogenesis, cyp17a1, *Seriola*

## Abstract

There is accumulating evidence that insulin-like growth factors (IGFs), primary mediators of somatic growth, play an important role in fish reproduction. Previously, we showed that IGF-1 and IGF-2 are expressed in the ovarian follicle cells of the yellowtail (*Seriola quinqueradiata*) during the vitellogenic phase, suggesting that IGFs may be involved in ovarian steroidogenesis. In this study, we examined the effects of IGF-1 and IGF-2 on gene expression and activity of steroidogenic enzymes in yellowtail ovary *in vitro*. IGF-1 and IGF-2 had no effect on mRNA levels of several steroidogenesis-related genes (*star*, *cyp11a1*, *hsd3b*, *cyp17a2*, and *cyp19a1*). However, both IGFs enhanced the transcription of *cyp17a1* in vitellogenic ovaries, although such up-regulation was not found in the ovary at the pre-vitellogenic stage. The stage-dependent effects of IGFs were correlated with changes in ovarian *cyp17a1* mRNA levels during the reproductive cycle: transcript abundances increased in conjunction with ovarian development. In addition, IGF-induced *cyp17a1* gene expression was significantly inhibited by wortmannin, suggesting that PI3 kinase plays an essential role in IGF-mediated ovarian steroidogenesis. Furthermore, IGF-1 and IGF-2 promoted the conversion of both progesterone and 17α-hydroxyprogesterone to androstenedione in vitellogenic ovaries, suggesting that both IGFs stimulated 17α-hydroxylase and C_17-20_ lyase activities. Taken together, these findings suggest that IGF-1 and IGF-2 act directly on follicle cells to stimulate steroid production through an increase in gene expression and enzymatic activity of *cyp17a1* via induction of PI3 kinase.

## 1 Introduction

Insulin-like growth factors (IGFs: IGF-1 and IGF-2), primary mediators of somatic growth, play essential roles in gonadal development and maturation in a wide variety of vertibrates. In mammals, IGFs have been shown to affect gonadal steroidogenesis, differentiation and proliferation of somatic cells in the gonads, and be involved in oocyte maturation (Bondy et al., 2006). IGF-1 has also been shown to potentiate the stimulatory effects of gonadotropins on steroid production and expression of steroidogenic enzymes in mammalian theca cells (Bondy et al., 2006). In teleosts, as in mammals, IGF-1 stimulates steroid production in the ovarian follicles. Furthermore, both IGF-1 and IGF-2 induce maturational competence and final oocyte maturation (Reinecke, 2010). In general, fish IGFs are primarily produced in the liver and exert their actions in various tissues to induce growth, proliferation, and differentiation of peripheral tissues via the blood stream (Reindl and Sheridan, 2012). However, IGFs are also expressed in parenchymal cells of numerous extrahepatic sites where they are thought to act via autocrine/paracrine mechanisms (Reindl and Sheridan, 2012; Wood et al., 2005). In fact, IGFs as well as their receptors are expressed in the ovary of teleosts, such as the zebrafish (*Danio rerio*) (Zhou et al., 2016), coho salmon (*Oncorhynchus kisutch*) (Maestro et al., 1997), common carp (*Cyprinus carpio*) (Mukherjee et al., 2006), and red seabream (*Pagrus major*) (Kagawa et al., 1995). Moreover, these ovarian IGFs are likely to be directly controlled by GH in the same manner as hepatic IGFs (Gioacchini et al., 2005; Berishvili et al., 2010). Therefore, ovarian IGFs are likely to be autocrine/paracrine regulators and play essential roles in the reproductive physiology of fish.

Although evidence regarding the possible role of IGF-2 is still lacking, several studies in fish have revealed the species-specific effects of IGF-1 on ovarian steroidogenesis. In the common carp (Paul et al., 2010) and white perch (*Morone americana*) (Weber et al., 2007), IGF-1 stimulated both testosterone and 17β-estradiol (E2) synthesis in ovarian follicles. In the coho salmon, IGF-1 inhibited basal testosterone production by isolated theca-interstitial layers, whereas it stimulated the production of both E2 and maturation inducing hormone by granulosa cell layers (Maestro et al., 1997). Similarly, IGF-1 only stimulated E2 production in ovarian follicles of the red seabream (Kagawa et al., 2003). Contrary to these steroidogenic actions, it was reported that IGF-1 had no effect on ovarian steroid production in the mummichog (*Fundulus heteroclitus*) (Negatu et al., 1998) and in the zebrafish (Nelson and Van Der Kraak, 2010a).

The species-specific actions of IGFs in fish steroidogenesis are likely to correspond to their expression patterns in ovaries because the expression patterns of IGF genes in ovaries also varied among fish (Higuchi et al., 2016). For example, levels of *igf-*1 mRNA in the ovaries of adult tilapia (*Oreochromis mossambicus*) (Schmid et al., 1999) and shi drum (*Umbrina cirrosa*) (Patruno et al., 2006) were much higher than those of *igf-*2 mRNA, whereas *igf-*2 mRNA was present at higher levels than *igf-1* in the rainbow trout (*Oncorhynchus mykiss*) (Lankford and Weber, 2010). In contrast, the expression of *igf-1* gene was considerably low or not detected in gilthead seabream (*Sparus aurata*) (Perrot et al., 2000), zebrafish (Nelson and Van Der Kraak, 2010b), and coho salmon ovaries (Yamamoto et al., 2011). Therefore, to understand the various steroidogenic actions of IGFs in the fish ovary, comparative studies regarding their gene expression and steroidogenic actions may be useful. In particular, autocrine/paracrine regulators are pivotal for the interactions between germ and somatic cells, as well as theca and granulosa cells in steroidogenesis. However, available information about the localization of IGFs in ovaries is still limited. Recently, we have shown that IGF-1 and IGF-2 in the yellowtail are produced in the theca and granulosa cells during the vitellogenic phase, suggesting that they are involved in the ovarian steroid production (Higuchi et al., 2016). In particular, the cellular localization of IGF-1 in the yellowtail (*Seriola quinqueradiata*) is distinct from that in other fish species studied so far, in which IGF-1 is mainly expressed in granulosa cells (Kagawa et al., 1995; Schmid et al., 1999; Perrot et al., 2000). The unique expression pattern of yellowtail IGF-1 may, thus, be indicative of a completely different function of IGF-1 in ovarian steroid production.

To gain a better understanding of the various physiological roles of IGFs in fish reproduction, we examined the regulatory roles of IGF-1 and IGF-2 for the gene expression and activity of steroidogenic enzymes in ovaries of our model fish species, the yellowtail, which is known as a multiple-spawner with an asynchronous-type ovary (Matsuyama et al., 1996). We also investigated the possible regulation of its steroidogenic enzymes through IGF receptors at different ovarian developmental stages.

## 2 Materials and methods

### 2.1 Fish and sampling procedures

All experiments were performed in accordance with the Guidelines for the care and use of live fish, Seikai National Fisheries Research Institute (SNFRI), Japan Fisheries Research and Education Agency (FRA). Yellowtail juveniles were captured from the wild and maintained for 2 years under natural water temperature and photoperiod conditions in square sea cages (5 m side, 5 m depth) at Goto station, SNFRI, FRA (Nagasaki, Japan). Fish were fed a commercial pellet diet (Hamachi special 15, Marubeni Nissin Feed, Tokyo, Japan) to satiation by hand each morning, 3 days/week.

For *in vitro* culture, ovaries at various developmental stages were sampled from yellowtail aged >2 years [approximately 6 kg in body weight (BW)] between January and April. The females were netted from cages, killed by decaptitation, and BW was measured. Ovarian tissues were removed and weighed to determine the gonadosomatic index [GSI = gonad weight (g) × 100/BW (g)]. A piece of ovary was fixed with Bouin's fixative for histological examination of the ovarian developmental stage, and other samples were placed in chilled Leibovitz's L-15 culture medium (Thermo Fisher Scientific Inc., Waltham, MA) supplemented with 0.1% bovine serum albumin (BSA, Sigma-Aldrich Inc., St. Louis, MO), 100 μg/ml streptomycin, 100 U/ml penicillin (Thermo Fisher Scientific Inc.), and 10 mM HEPES adjusted to pH 7.4 for ovarian culture. Developmental stages of ovaries were classified into the perinucleolar stage (Pn), yolk vesicle stage (Yv), primary yolk stage (Py), secondary yolk stage (Sy), and tertiary yolk stage (Ty), according to the most advanced types of oocytes found, as described previously (Higuchi et al., 2016).

To investigate the distribution of IGF receptor gene expression among tissues, four females of age >2 years were sampled on January 2014 (Higuchi et al., 2016). The brain, pituitary, gill, heart, liver, kidney, stomach, spleen, muscles and ovary were collected, immediately placed in RNAlater (Ambion, Austin, TX), and stored at -30 °C until analysis. To investigate changes in *cyp17a1* gene expression associated with ovarian development, we sampled 88 females aged >2 years at various stages of ovary development between July 2012 and May 2013 (Higuchi et al., 2016). The ovary samples were placed in RNAlater, and stored at -30 °C.

### 2.2 Ovarian culture

Human recombinant IGF-1 and IGF-2 were purchased from Bachem AG (Bubendorf, Switzerland) and Sigma-Aldrich, respectively. IGFs were dissolved at 50 μM in phosphate-buffered saline (PBS) with 0.1% BSA. Wortmannin (Wort) was purchased from Cayman Chemical (Ann Arbor, MI), and dissolved at 10 mM in DMSO. Progesterone (P) and 17α-hydroxyprogesterone (17-P) were purchased from Sigma-Aldrich. The steroids were first dissolved in absolute ethanol at 1 mg/ml, then diluted in L-15 medium to 10 μg/ml. All stock solutions of chemicals were dissolved directly in the culture medium at less than 0.1% (v/v) vehicle. Ovarian tissue culture was performed by the method widely used in fish species (Kagawa et al., 2003; Weber et al., 2007; Luckenbach et al., 2011; Yamamoto et al., 2011; Yang et al., 2015) with some modifications. Briefly, ovaries were cut into approximately 40 mg pieces. One piece was transferred into a well of a 48-well polystyrene culture plate containing 0.5 ml of L-15 medium. After 1 h of pre-incubation in L-15 medium without any additives at 20 °C, the medium was removed and replaced with either fresh medium alone (control) or medium containing IGF-1 (1, 10 and 100 nM) or IGF-2 (1, 10 and 100 nM) and incubated over 8–48 h. Wort (1 and 10 μM), P (100 ng/ml) or 17-P (100 ng/ml) were also tested in combination with IGFs. After incubation, the sampled ovarian fragments were stored in RNAlater at -30 °C for RNA isolation. The culture medium was collected and frozen at -80 °C for steroid assays. All incubations were performed in triplicate wells per treatment. In addition, each experiment was repeated using 2–4 different ovaries to confirm the reproducibility.

### 2.3 RNA isolation and reverse transcription

Total RNA was extracted from the cultured ovaries using ISOGEN II (NIPPON GENE, Toyama, Japan), and treated using TURBO DNase (Ambion) according to the manufacturer's protocol. One μg of total RNA, quantified using a NanoDrop (ND-1000, Thermo Scientific Inc.), was reverse-transcribed using the Omniscript RT kit (QIAGEN GmbH, Dusseldorf, Germany), after priming with random hexamers (QIAGEN GmbH).

For the across-stage comparisons of transcript levels, mRNA was further isolated from total RNA samples to mitigate issues associated with comparing ovarian follicles during different stages of oogenesis, which may be dramatically different in size and RNA composition (Yamamoto et al., 2011). mRNA was isolated from 60 μg of total RNA/sample using the Poly(A) Purist MAG kit (Ambion), and 50 ng of mRNA was reverse-transcribed as described above. As *in vitro* culture experiments were done with ovaries at the same stage, total RNA was used for cDNA synthesis.

### 2.4 RT-PCR and real-time quantitative PCR

RT-PCR was used to study tissue distribution of IGF receptors (*igf1ra*, *igf1rb*, *igf2r*) mRNA in yellowtail females, and the PCR products were then electrophoresed and visualized in 2.0% agarose gel containing ethidium bromide. The primers for RT-PCR were designed and synthesized by Greiner Bio One International GmbH (Kremsmunster, Austria) (Table 1).

**Table 1:** RT-PCR primer sequences for targeted genes and PCR product sizes.

Targeted gene	Primer/probe sequence (5′-3′)		Product size (bp)
*igf1ra*	Forward:	AGGGCAATCTGGACATCAAC	300
	Reverse:	ACATGCAGAGTTTGGGGTTC	
*igf1rb*	Forward:	GCATATCAACATCCGCAGAG	445
	Reverse:	TTGCCAGGTCAGTTTGATCC	
*igf2r*	Forward:	ATCACCTTCACCTGTCCATC	430
	Reverse:	CATCTGTCACCGTCTGGGTA	
*actinb*	Forward:	GATGAAGCCCAGAGCAAGAG	432
	Reverse:	GAAGGAGTAGCCACGCTCTG	

Abundance of gene transcripts was determined using the Taqman probe method in quantitative real-time PCR assays. Gene-specific primers and probes of yellowtail steroidogenesis-related genes (*star*, *cyp11a1*, *hsd3b*, *cyp17a1*, *cyp17a2*, *cyp19a1*) and β-actin (*actinb*) were used as described previously by Higuchi et al. (2017), and purchased from Integrated DNA Technologies (Coralville, IA) (Table 2). Assays were run on a Light Cycler 480 (Roche Diagnostics, Mannheim, Germany) in 96-well plates (Roche Diagnostics), using standard cycling conditions: 95 °C for 10 min, followed by 45 cycles of 95 °C for 10 s and 58 °C for 30 s. Reaction volumes (10 μl) contained 2.5 μl of cDNA template (reverse transcription products diluted 10-fold in distilled water), 0.5 μM of the forward and reverse primers, 0.2 μM of probe, 5 μl of FastStart Essential DNA Probes Master (2 ×) (Roche Diagnostics), and 2 μl of distilled water. For each PCR, a standard curve from serial dilution of a plasmid containing a partial cDNA sequence of a target gene was constructed. The standard sets of 7 points ranged from 1 × 10^7^ to 1 × 10^1^ copies, and were prepared by 10 × serial dilution. Technical duplicates were run for all experimental samples and standards. For ovarian culture experiments, results for each target gene were normalized using *actinb* as a reference. The mean value of the 8 h control or 0 nM IGFs control were set to 1 to improve the presentation of results. For the across-stage comparisons of *cyp17a1* mRNA levels, results were not normalized using a reference gene because there was no reference gene found to be stable among the different stage ovaries.

**Table 2:** Quantitative PCR primer and probe sequences for targeted genes, PCR product sizes, and mean cycle threshold (C_T_) values for all cDNA samples measured.

Targeted gene	Primer/probe sequence (5′-3′)		Product size (bp)	Mean C_T_
*cyp17a2*	Forward:	GGGAGGACTGGTGGACATTTAC	85	31.3
	Probe:	FAM-CCTGGATGAAGGTCTTTCCTAACAAGTCTCTGA-IBFQ		
	Reverse:	TCTGACAGTGATACAGTCCTTCAGTTT		
*actinb*	Forward:	ACCCTGTCCTGCTCACAGAG	137	20.5
	Probe:	FAM-AGATGACCCAGATCATGTTCGAGA-IBFQ		
	Reverse:	ACCAGAGGCATACAGGGACA		

Intra-assay coefficients of variation (CVs) were determined by repeated measurement (*n* = 8) of standard samples (yellowtail cDNA from ovary) within each assay. For assays of *star*, *cyp11a1*, *hsd3b*, *cyp17a1*, *cyp17a2* and *cyp19a*, intra-assay CVs were 0.3, 2.0, 3.1, 4.3, 6.8 and 1.4%, respectively. For each experiment, all samples were assayed at the same time, obviating inter-assay variability.

### 2.5 Measurement of androstenedione concentrations in incubation media

The concentration of androstenedione in the media was measured by time-resolved fluoroimmunoassay following the methods by Yamada et al. (1997). In brief, steroids were extracted from 100 μl culture medium with diethyl ether, the ether was evaporated and the resultant extracts were reconstituted in 100 μl of assay buffer (0.05 M Tris, 0.9% NaCl, 0.5% BSA, 0.05% NaN3, 0.01% Tween 40, 20 μM diethylenetriamine-N, N, N′, N″, N″-pentaacetic acid, pH 7.75). Wells of 96-well plates were coated with BSA-conjugated androstenedione prepared by a method reported elsewhere (Asahina et al., 1995). The steroid extracts were incubated with an antiserum against androstenedione (FKA-138, Cosmobio, Tokyo, Japan) in the coated 96-well plates. After incubation at 20 °C for 4 h, the plates were washed with PBS containing 0.05 % Tween 20 (PBST) three times. Europium (Eu)-labeled goat anti-rabbit IgG (AD0105, Perkin-Elmer, Waltham. MA) was added to each well and incubated at 20 °C for 1 h. After washes, an enhancement solution (Perkin-Elmer) was added and the fluorescence signals from dissociated Eu were measured using an Infinite F200 plate reader (TECAN, Grodig, Austria). Intra-assay CV for standard samples in the same plate were 5.56% (*n* = 3). For each experiment, all samples were assayed at the same time, obviating inter-assay variability.

### 2.6 Statistical analysis

Data are presented as the mean ± standard error of the mean (SEM). Time course data from ovarian culture experiments were analyzed using two-way analysis of variance (ANOVA) with treatment and time as independent variables, and where significant differences were observed, Tukey's multiple comparison tests were conducted. The across-stage gene expression data and IGF concentration-response data (with or without Wort, P and 17-P) were subjected to one-way ANOVA followed by Tukey's multiple comparison tests. Significant differences in *cyp17a1* mRNA levels between IGF-treated and control samples in ovaries at different stages were analyzed using Student's *t*-test. Statistical analyses were performed using Prism 6.0 (GraphPad Software, San Diego, CA).

## 3 Results

### 3.1 Effects of IGF-1 and IGF-2 on expression of ovarian steroidogenesis-related genes

**Figure 1:**
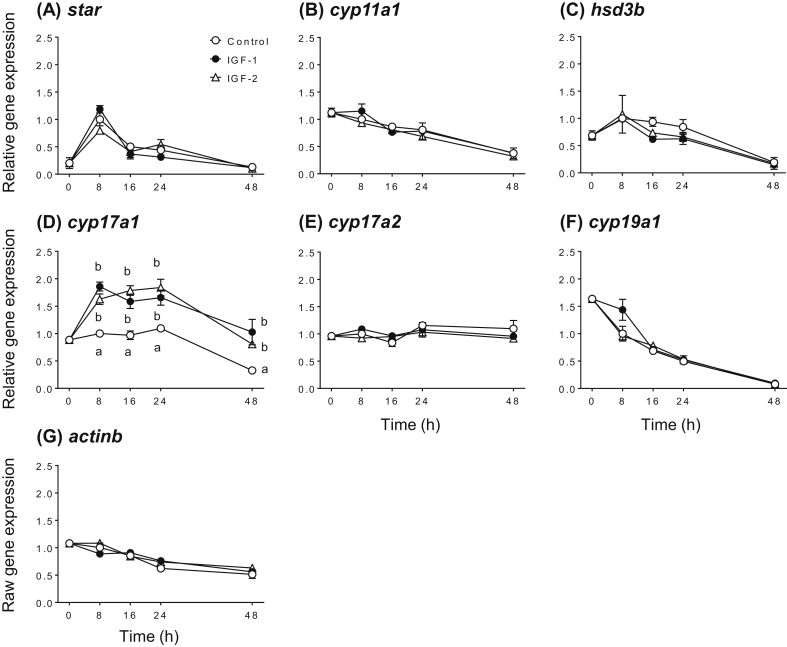
Effects of IGF-1 and IGF-2 on mRNA levels of steroidogenesis-related genes, *star* (A), *cyp11a1* (B), *hsd3b* (C), *cyp17a1* (D), *cyp17a2* (E) and *cyp19a1* (F), and reference gene, *actinb* (G) in yellowtail ovaries at the tertiary yolk stage. Ovarian fragments were incubated in 0.5 ml culture medium containing IGF-1 or IGF-2 (100 nM) for 8–48 h at 20 °C. Quantitative PCR data for genes of interest were normalized to *actinb* mRNA levels, whereas *actinb* data were not normalized to another gene. The data represent mean ± SEM (*n* = 3). Significant differences between IGF treatment and control groups at each time point are indicated by different letters (*P* < 0.05, two-way ANOVA followed by Tukey's multiple comparison test). The figure shows a representative result of replicates from at least three experiments.

To examine effects of IGF-1 and IGF-2 on expression of ovarian steroidogenesis-related genes, IGFs time-course experiments were conducted using yellowtail ovary at the TY stage. The transcript levels of steroidogenesis-related genes showed clear responses to time-course treatment with IGF-1 and IGF-2 (Figure 1). Transcript abundance for *cyp17a1* was significantly increased in ovarian fragments treated with both IGFs by 8 h, and remained elevated above controls at all time points (*P* < 0.05). IGF-1 and IGF-2 had no effect on mRNA levels of other steroidogenesis-related genes over the time course investigated (*P* > 0.05).

**Figure 2:**
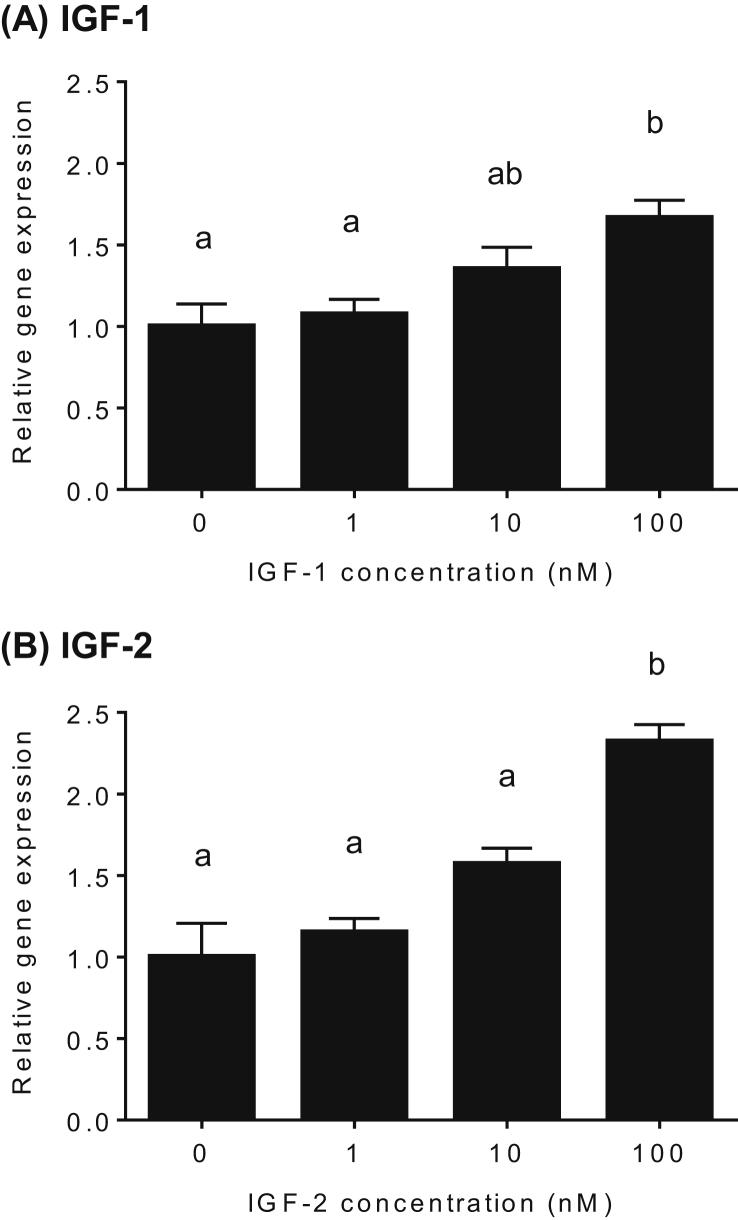
Effects of different concentrations of IGF-1 (A) and IGF-2 (B) on *cyp17a1* mRNA levels in yellowtail ovaries at the tertiary yolk stage. Ovarian fragments were incubated in 0.5 ml culture medium containing different doses of IGF-1 or IGF-2 (0, 1, 10, or 100 nM) for 8 h at 20 °C. Quantitative PCR data for *cyp17a1* genes were normalized to *actinb* mRNA levels. The data represent mean ± SEM (*n* = 3). Different letters indicate statistically significant differences among different IGF doses (*P* < 0.05, one-way ANOVA followed by Tukey's multiple comparison test). The figure shows a representative result of replicates from at least three experiments.

Transcription of *cyp17a1* that exhibited significant upregulation at 8 h in the time-course experiments was further assessed with different concentration of IGFs using ovary tissue at the TY stage (Figure 2). Transcript abundance for the *cyp17a1* gene increased in a concentration-dependent manner, reaching approximately 2-fold maximum elevation when treated with 100 nM IGFs (*P* < 0.05).

### 3.2 Stage-dependent effects of IGF-1 and IGF-2 on ovarian *cyp17a1* gene expression and developmental changes in ovarian *cyp17a1* mRNA levels

**Figure 3:**
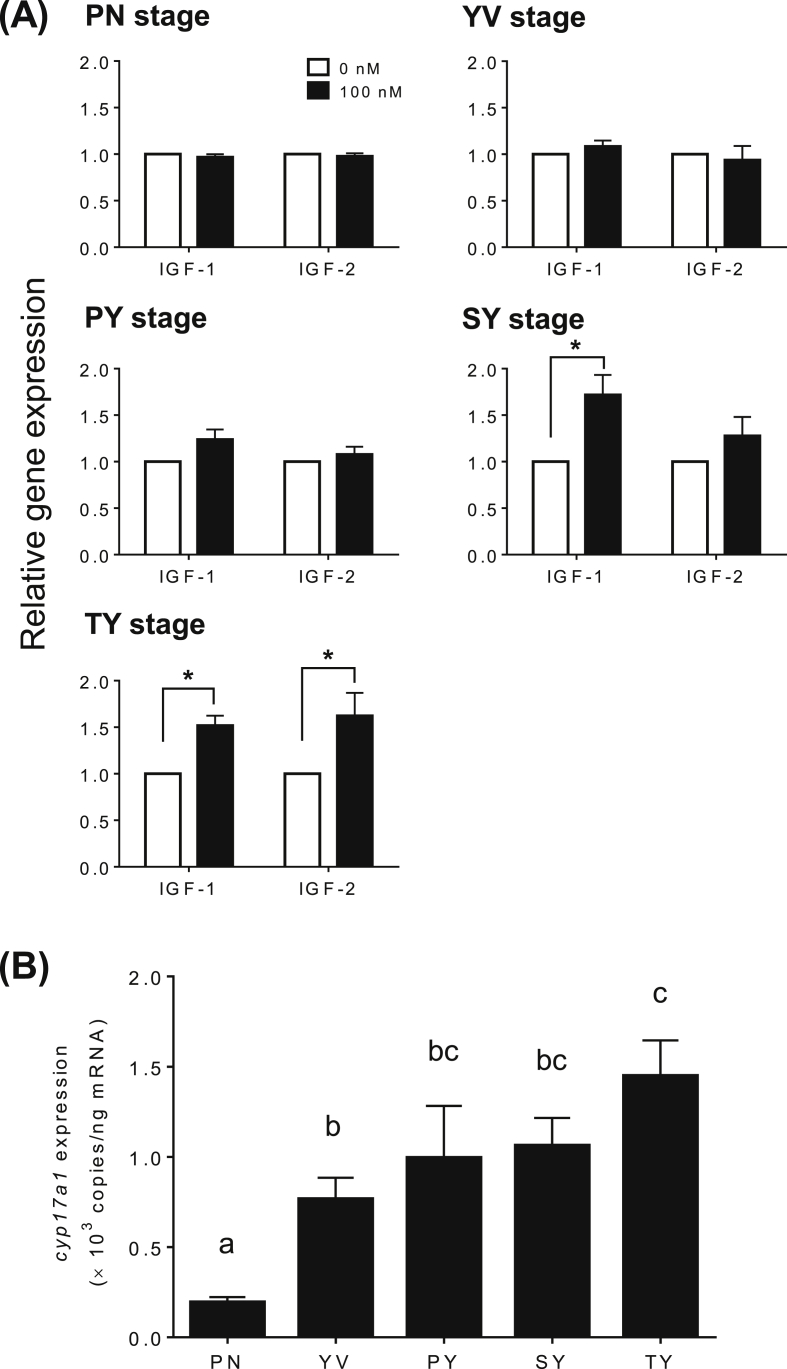
Stage-dependent effects of IGF-1 and IGF-2 on *cyp17a1* mRNA levels in yellowtail ovaries. Developmental stages of ovaries were classified into the perinucleolar stage (Pn), yolk vesicle stage (Yv), primary yolk stage (Py), secondary yolk stage (Sy), and tertiary yolk stage (Ty), according to the most advanced types of oocytes found. (A) Effects of IGF-1 and IGF-2 on *cyp17a1* mRNA levels in ovaries at different developmental stages in female yellowtail fish. Ovarian fragments at different developmental stages were incubated in 0.5 ml culture medium containing IGF-1 or IGF-2 (100 nM) for 8-h at 20 °C. The data represent mean ± SEM of replicates from 3 or 4 different ovaries (*n* = 3 or 4). Asterisks denote significant differences between IGF-treated and control groups (*P* < 0.05, Student's *t*-test). (B) Changes in levels of transcripts of *cyp17a1* gene during ovarian development. The gene expression was absolutely quantified using the standard curve constructed from serial dilution of a plasmid containing a partial cDNA sequence of *cyp17a1* gene. Quantitative PCR data for *cyp17a1* gene were not normalized using a reference gene. The data represent means ± SEM (*n* = 48, Pn; *n* = 10, Yv; *n* = 3, Py; *n* = 8, Sy; *n* = 12, Ty). Different letters indicate statistically significant differences at different stages of ovarian development (*P* < 0.05, one-way ANOVA followed by Tukey's multiple comparison test).

To examine reproductive stage-dependent effects of IGF-1 and IGF-2 on *cyp17a1* gene expression, ovarian fragments at various developmental stages (PN, YV, PY, SY, or TY stages) were incubated for 8 h with 100 nM of IGF-1 or IGF-2. The cultures were conducted using 3 or 4 different females at each developmental stage (GSI, 0.55 ± 0.03 at PN stage; 0.61 ± 0.05 at YV stage; 0.98 ± 0.08 at PY stage; 1.88 ± 0.24 at SY stage; 2.81 ± 0.10 at TY stage). Transcript abundance for *cyp17a1* were significantly increased by IGF-1 relative to control in SY and TY stage ovaries (*P* < 0.05), but not in PN, YV and PY stage ovaries (Figure 3A). IGF-2 significantly stimulated *cyp17a1* mRNA levels only in TY stage ovaries (*P* < 0.05, Figure 3A).

Ovarian *cyp17a1* transcript levels were measured at the various developmental stages. The expression of ovarian *cyp17a1* was lowest at the PN stage, thereafter *cyp17a1* mRNA levels exhibited their first significant increase at the YV stage, and reached their maximum levels at the TY stage (Figure 3B).

### 3.3 RT-PCR analysis of tissue distribution of IGF receptor gene expression

**Figure 4:**
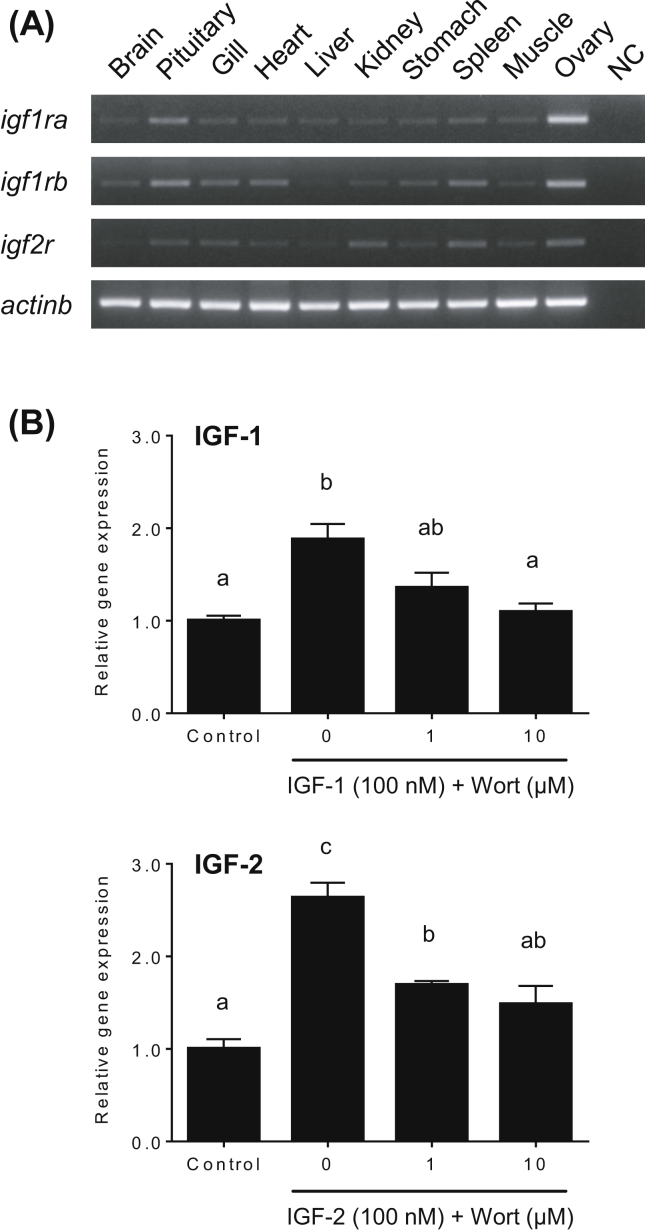
Tissue distribution of transcripts for IGF receptor genes (*igf1ra*, *igf1rb*, and *igf2r*) in the yellowtail and effects of the PI3 kinase inhibitor, Wortmannin (Wort), on IGF-induced *cyp17a1* mRNA levels in yellowtail ovaries at the tertiary yolk stage. (A) cDNA from various tissues (brain, pituitary, gill, heart, liver, kidney, stomach, spleen, muscles, and ovary) of females aged >2 years were used for RT-PCR. *Actb* was used as an endogenous reference. Lane NC is the negative control containing no cDNA template. The full, non-adjusted images are available as a supplementary material. (B) Ovarian fragments were incubated in 0.5 ml culture medium containing IGF-1 or IGF-2 (100 nM) with Wort (0, 1, or 10 μM) for 8-h at 20 °C. Quantitative PCR data for *cyp17a1* genes were normalized to *actinb* mRNA levels. The data represent mean ± SEM (*n* = 3). Different letters indicate statistically significant differences among different experimental groups (*P* < 0.05, one-way ANOVA followed by Tukey's multiple comparison test). The figure shows a representative result of replicates from different two experiments.

The expression of *igf1ra*, *igf1rb* and *igf2r* was detected in all tissues investigated, including the ovary (Figure 4A).

### 3.4 Effects of PI3 kinase inhibitor on IGF-induced *cyp17a1* gene expression

To examine whether PI3 kinase activation was necessary for IGF-induced *cyp17a1* gene expression, TY stage ovary fragments were pre-incubated for 2-h with increasing concentrations of Wort (0, 1 or 10 μM), followed by incubation with IGF-1 or IGF-2 (100 nM) for a further 8-h. Wort, at its increasing concentrations, significantly inhibited both IGF-1 and IGF-2-stimulated *cyp17a1* gene expression almost in a dose-dependent manner (*P* < 0.05, Figure 4B).

### 3.5 Effects of IGF-1 and IGF-2 on 17α-hydroxylase and C_17-20_ lyase activities

**Figure 5:**
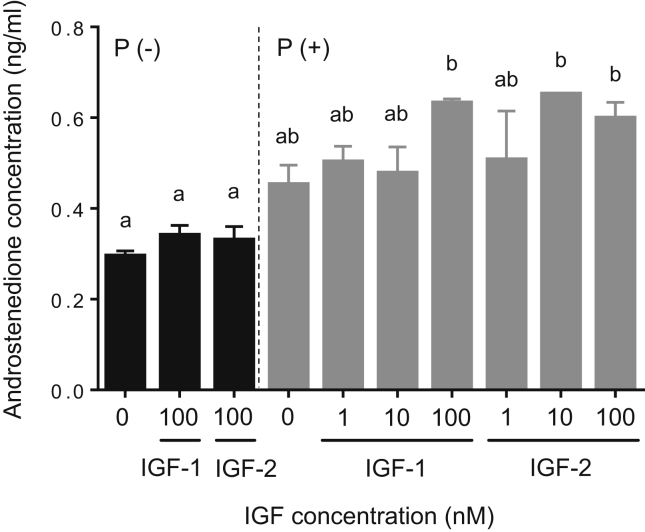
Effects of IGF-1 and IGF-2 on 17α-hydroxylase and C_17-20_ lyase activities by tertiary yolk stage ovaries in the yellowtail. Ovarian fragments were incubated in 0.5 ml culture medium containing different doses of IGF-1 or IGF-2 (0, 1, 10, or 100 nM) with or without progesterone (P, 100 ng/ml) for 24-h at 20 °C. The data represent mean ± SEM (*n* = 3). Different letters indicate statistically significant differences among different experimental groups (*P* < 0.05, one-way ANOVA followed by Tukey's multiple comparison test). The figure shows a representative result of replicates from at least three experiments.

**Figure 6:**
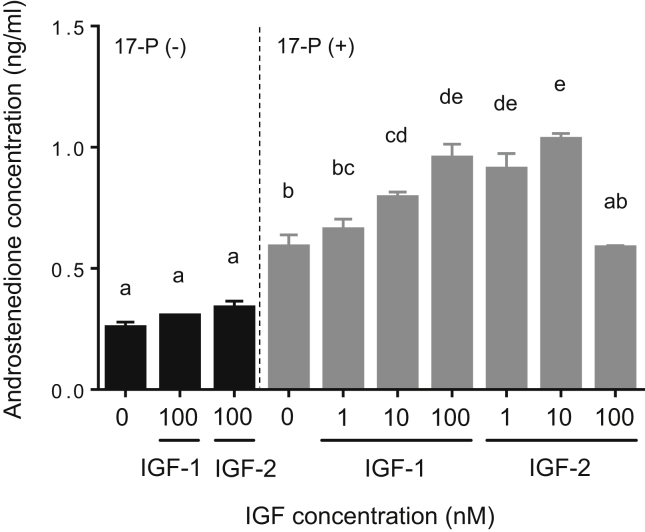
Effects of IGF-1 and IGF-2 on C_17-20_ lyase activity in tertiary yolk stage ovaries of yellowtail. Ovarian fragments were incubated in 0.5 ml culture medium containing different doses of IGF-1 or IGF-2 (0, 1, 10, or 100 nM) with or without 17α-hydroxyprogesterone (17-P, 100 ng/ml) for 24-h at 20 °C. The data represent mean ± SEM (*n* = 3). Different letters indicate statistically significant differences among different experimental groups (*P* < 0.05, one-way ANOVA followed by Tukey's multiple comparison test). The figure shows a representative result of replicates from at least three experiments.

To examine whether IGF-1 and IGF-2 stimulate 17α-hydroxylase and C_17-20_ lyase activities, TY stage ovary fragments were incubated with IGFs in the absence or presence of P or 17-P as precursor substrate for 24-h, and then androstenedione content in the medium was measured. IGF-1 and IGF-2 at 100 nM in the absence of P or 17-P did not affect androstenedione production (Figures 5 and 6). Androstenedione production, however, increased significantly after high doses of IGF-1 (100 nM) and IGF-2 (10 and 100 nM) in the presence of P in the medium (*P* < 0.05, Figure 5). IGF-1 had stimulatory effects on androstenedione production in a concentration-dependent manner when 17-P was present in the incubation medium (*P* < 0.05, Figure 6). Moreover, IGF-2 significantly elevated androstenedione production with addition of 17-P to the medium (*P* < 0.05), but the highest dose of IGF-2 (100 nM) did not promote such production (Figure 6).

## 4 Discussion

In the present study, we demonstrated that IGF-1 and IGF-2 could stimulate only *cyp17a1* gene expression in the yellowtail ovary *in vitro*. However, although information is limited regarding the effects of IGF-2 on ovarian steroidogenesis, the steroidogenic actions of ovarian IGF-1 detected in this study differ from those of other reports involving other fish species. For example, in the common carp, IGF-1 enhanced *cyp19a1* gene expression in isolated granulosa cells, although the administration of IGF-1 stimulated basal testosterone and E2 production in vitellogenic follicles (Paul et al., 2010). Moreover, the release of E2 from the ovaries was elevated through an increase in *cyp19a1* mRNA levels after IGF-1 treatment in several Salmonidae species, whereas testosterone production is inhibited (Maestro et al., 1997; Nakamura et al., 2016). Furthermore, in red seabream ovary, IGF-1 promoted only the conversion of testosterone to E2 by stimulating aromatase activity and *cyp19a1* gene expression (Kagawa et al., 2003). Interestingly, red seabream IGF-1 proteins were found only in the granulosa cell layers at different developmental stages (Kagawa et al., 1995). This cellular localization of IGF-1 in the ovary is strongly correlated with the steroidogenic action, because the theca cells (potential sites expressing *cyp17a1* gene) supply testosterone to the granulosa cells that express *cyp19a1* and produce E2 in vitellogenic fish (Lubzens et al., 2010). In contrast, yellowtail IGF-1 was mainly expressed in the theca cells (Higuchi et al., 2016), and then stimulated gene expression of *cyp17a1* and enzymatic activities, i.e., 17α-hydroxylase/C_17-20_ lyase. In addition, IGF-1 had no effect on *cyp19a1* gene expression and aromatase activity in yellowtail ovaries (data not shown). Therefore, the steroidogenic actions of IGF-1 are, in part, likely to be derived from differences in IGF expression among fish species. Meanwhile, autocrine/paracrine regulators including IGFs are secreted proteins that can diffuse and interact between the different gonadal cell types involved in steroid production. This fact suggests that cellular localization of IGF receptors may be also correlated with the different steroidogenic actions of fish IGFs. In future, further studies are needed to examine the localization of IGF receptors in ovaries of the yellowtail as well as other fish species.

The transcript levels of steroidogenesis-related genes except for *cyp17a2* showed a slight decline between 24 and 48 h, regardless of the presence of IGFs. Luckenbach et al. (2011) identified that expression of genes associated with cell survival were decreased over time in culture of coho salmon ovary. Although we did not determine the expression levels of cell survival-related genes in the cultured yellowtail ovaries, transcript levels of *actinb* was tended to be decreased from 24 h onward, suggesting the possibility of a decline in at least cell activity of the ovarian follicles. Therefore, these results could probably explain the decreasing expression levels observed for steroidogenesis-related genes.

In teleosts, as in mammals, there are two types of IGF receptor; type 1 (IGF-1R) and type 2 (IGF-2R) (Caruso and Sheridan, 2011). IGFs binding to IGF-1R leads to activation of tyrosine kinase, resulting in phosphorylation of insulin receptor substrate (IRS) which subsequently activates downstream signaling molecules including the PI3 kinase and MAP kinase signaling cascades (Backer et al., 1992a, 1992b; Chuang et al., 1993). Although it is not certain how IRS associates with PI3 kinase in inducing ovarian steroidogenesis in fish, PI3 kinase existed in carp ovarian follicle cells and can be activated by IGF-1 for steroid production (Paul et al., 2013). In the present study, ovarian gene expression of IGF-1R (*igf1ra* and *igf1rb*) and IGF-2R was demonstrated and, furthermore, a PI3 kinase inhibitor, Wort, blocked IGF-1-and IGF-2-induced *cyp17a1* expression in yellowtail ovarian follicles. These findings suggest that PI3 kinase plays an essential role in IGF-mediated steroid production, and the actions of both IGF-1 and IGF-2 may be mediated through activation of IGF-1R but not IGF-2R in yellowtail ovarian follicles. In general, the affinity of IGF-1R for IGF-1 is typically greater than that for IGF-2, and the affinity of IGF-2R for IGF-2 is higher than that for IGF-1 (Jones and Clemmons, 1995; Méndez et al., 2001; Hawkes and Kar, 2004). However, steroidogenic actions of mammalian IGF-1 and IGF-2 in granulosa, theca, and luteal cells are mediated via IGF-1R but not IGF-2R (Adashi et al., 1990; Blakesley et al., 1996; Willis et al., 1998). Moreover, ligand binding assays performed with zebrafish cells indicated that IGF-1 and IGF-2 bound to IGF-1R with similar affinities (Pozios et al., 2001). In future studies, it would be useful to examine the affinity of IGF-1 and IGF-2 for yellowtail IGF receptors for improved understanding of regulatory mechanisms involving IGF-1 and IGF-2 in ovarian steroid production.

In the present study, both IGFs significantly promoted the conversion of P and 17-P to androstenedione, suggesting that IGFs stimulate both 17α-hydroxylase and C_17-20_ lyase activities. In teleosts, unlike other vertebrates, two cytochrome P450c17 enzymes (P450c17-I and -II encoded by *cyp17a1* and *cyp17a2* genes, respectively) have been isolated and characterized (Zhou et al., 2007; Su et al., 2015). It has been demonstrated that P450c17-I exhibits both 17α-hydroxylase and C_17-20_ lyase activities, whereas P450c17-II is responsible for only 17α-hydroxylase activity. Therefore, the enhancement of both 17α-hydroxylase and C_17-20_ lyase activities by IGFs in the yellowtail ovary seems to be consistent with IGF-induced gene expression of *cyp17a1*. In addition, our previous study showed that both IGFs mRNA levels in yellowtail ovaries were elevated during the secondary oocyte growth phase, and then IGF-1 and IGF-2 proteins were produced in the follicle cell layers (Higuchi et al., 2016). Taken together, these findings suggest that ovarian IGF-1 and IGF-2 are potential autocrine/paracrine regulators in yellowtail ovaries, and act directly on follicle cells to stimulate steroid production through an increase in gene expression and enzymatic activity of *cyp17a1*. On the other hand, a high dose of IGF-2, but not IGF-1, did not affect the conversion of 17-P to androstenedione in the TY stage ovary of the yellowtail. The underlying mechanisms involving IGF-2, at this point, remain unclear. Further studies are needed to clarify the detailed mechanisms.

In conclusion, we have shown that IGF-1 and IGF-2 stimulate *cyp17a1* gene expression and the conversion of P to androstenedione (17α-hydroxylase and C_17-20_ lyase activities) in the ovary of the yellowtail. Moreover, the actions of both IGF-1 and IGF-2 are likely to be mediated via IGF-1R but not IGF-2R. To date, the stimulatory effects of IGF-1 on *cyp19a1* gene expression and/or aromatase activity have been reported in ovaries of several fish species (Kagawa et al., 2003; Paul et al., 2010) but, to the best of our knowledge, inductions of ovarian *cyp17a1* gene expression and 17α-hydroxylase/C_17-20_ lyase activities by IGF-1 and IGF-2 were demonstrated here for the first time in teleosts. Although IGF-1 acted synergistically with luteinizing hormone to increase expression of *cyp17* in human ovary, IGF-1 alone did not stimulate the expression of *cyp17* (Young and McNeilly, 2010). These results suggest that IGFs play different biological roles in ovarian steroid production among fish species, as well as mammals. Recently, gonad-specific expression of a novel IGF subtype, IGF-3, has been discovered in teleosts (Wang et al., 2008; Song et al., 2016) and amphibians (NP_001082137.1, NP_001120418.1). Additionally, IGF-3 is involved in mediation of ovarian steroidogenesis in the tilapia (Li et al., 2012) and oocyte maturation in the zebrafish (Li et al., 2015). However, the expression of IGF-3 was stimulated by gonadotropins (follicle-stimulating hormone) (Nóbrega et al., 2015), unlike that of IGF-1 and IGF-2, which were mainly regulated by GH (Berishvili et al., 2010; Yang et al., 2015). Further studies regarding regulatory mechanisms involving IGF ligands, including IGF-3 by GH and/or gonadotropins, will be important to better understand the potential roles of IGF signaling in reproduction of yellowtail fish.

## Declarations

### Author contribution statement

K. Higuchi: Conceived and designed the experiments; Performed the experiments; Analyzed and interpreted the data; Wrote the paper.

Y. Kazeto: Conceived and designed the experiments; Analyzed and interpreted the data; Wrote the paper.

Y. Ozaki: Analyzed and interpreted the data.

D. Izumida: Performed the experiments.

T. Hotta, K. Soyano and K. Gen: Contributed reagents, materials, analysis tools or data.

### Funding statement

Kentaro Higuchi was supported by 10.13039/501100001691Japan Society for the Promotion of Science (JP) (17K15316).

### Competing interest statement

The authors declare no conflict of interest.

### Additional information

No additional information is available for this paper.

## Appendix A Supplementary data

Higuchi et al. _spl_2020_spl_ 200607 supplementary Fig Higuchi et al. _spl_2020_spl_ 200607 supplementary Fig
