# Supplementary material for: Insulin-like growth factors 1 and 2 regulate gene expression and enzymatic activity of cyp17a1 in ovarian follicles of the yellowtail, Seriola quinqueradiata
Source: Heliyon. 2020 Jun 10;6(6):e04181. doi: 10.1016/j.heliyon.2020.e04181 (PMC7298419; doi:10.1016/j.heliyon.2020.e04181)
Supplement: Higuchi et al. _spl_2020_spl_ 200607 supplementary Fig [file mmc1.pdf]

Supplementary files

Original electrophoretic diagram for Figure 4

igf1ra
300 bp →

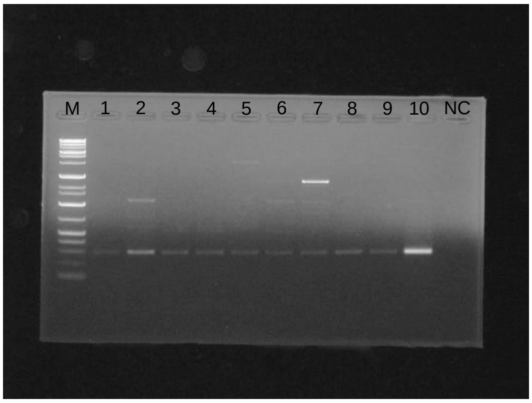

igf1rb
445 bp →

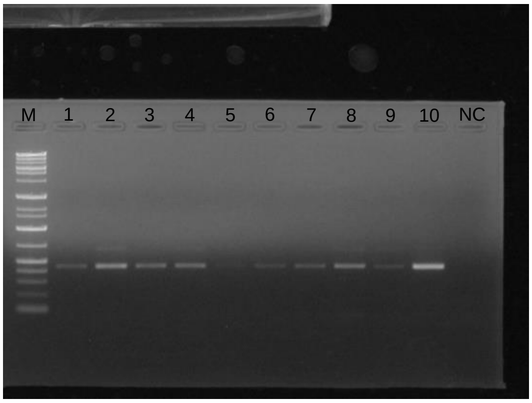

igf2r
430 bp →

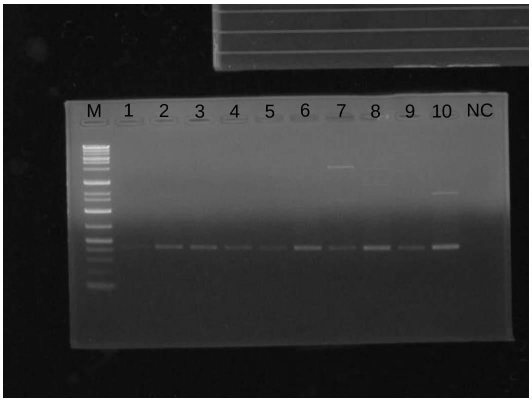

actinb
432 bp →

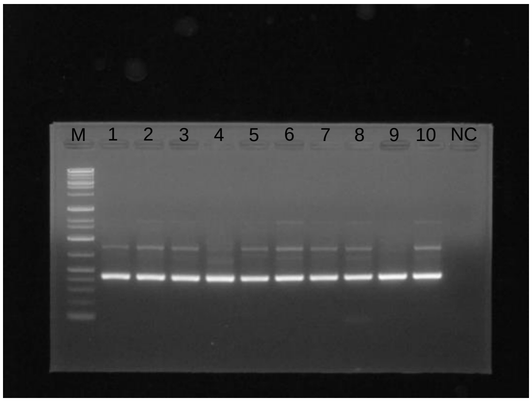

1: Brain
2: Pituitary
3: Gill
4: Heart
5: Liver
6: Kidney
7: Stomach
8: Spleen
9: Muscle
10: Ovary
NC: Negative control containing no cDNA template
